# Trends and Shifts in Swedish Telemedicine Consultations During the Pre–COVID-19, COVID-19, and Post–COVID-19 Periods: Retrospective Observational Study

**DOI:** 10.2196/60294

**Published:** 2025-05-16

**Authors:** Adaora Uloma Asomugha, Annamaria Pakai

**Affiliations:** 1 Doctoral School of Health Sciences, University of Pécs, Vörösmarty M u 4, Pécs, 7621, Hungary, 36 2818544910

**Keywords:** telemedicine, COVID-19, health care consultation, Sweden, digital health, patient behavior, video consultation, telehealth trends

## Abstract

**Background:**

In recent times, the telemedicine landscape has changed dramatically; it serves as a bridge, connecting health care providers and patients, especially during challenges such as the recent COVID-19 pandemic.

**Objective:**

This study seeks to explore the Swedish telemedicine landscape in terms of primary patient symptoms for teleconsultation and the patterns of telemedicine use in the periods before COVID-19, during COVID-19, and after COVID-19, including the primary care use dynamics with respect to the teleconsultations done.

**Methods:**

Secondary data was used in this observational retrospective study. The study population consisted of Swedish residents who had online telemedicine consultations. Telemedicine consultations were divided by text and video delivery; the period of analysis ranged from November 2018 to June 2023. The statistical methods used for the data analysis were descriptive analysis, 2-way cross tabulation, and a generalized linear model.

**Results:**

During the pandemic, the number of teleconsultations concerning general, unspecified symptoms increased in comparison to the other analyzed symptoms, signaling a change in care-seeking behavior under epidemiological pressure. General health-related issues were the most pronounced symptom across all periods: 186.9 of 1000 consultations before COVID-19, 1264.6 of 1000 consultations during COVID-19, and 319.2 of 1000 consultations after COVID-19. There was no significant main effect of COVID-19 period on the number of telemedicine consultation meetings (*F*_2_=1.653; *P*=.38). The interaction effect between delivery type and period was statistically significant (*F*_2_=14.723; *P*<.001).

**Conclusions:**

The findings are in favor of the COVID-19 pandemic having had a considerable effect on telemedicine use. Telemedicine could subsequently be used more often for general health consultations and acute conditions. Video consultations were more prominent because of the importance of bidirectional communication. The study suggests that there was a transformation of patterns of demand for health care; there is a necessity for health care systems to respond to these changes.

## Introduction

Despite it's recent popularity, the idea of telemedicine has it's roots in the early twentieth century [1,2]. Early on, telemedicine referred to the use of technologies like radio to enable patient access to medical care in remote locations. However, with the advancement of technology, telemedicine has evolved and has been optimized to use sophisticated platforms that incorporate advanced computing and communication technologies [3,4]. Today, the world is in dire need of telemedicine, which can bridge the gap between patients and health care providers and has become a realistic alternative to the traditional medical consultation [5]. Studies have shown that telemedicine is effective in providing timely interventions at reasonable cost; for instance, telemedicine substantially improved health outcomes and reduced hospitalization rates among patients with chronic illness [6-8].

Telemedicine has recently gained even more prominence and is being used more and more. For instance, the World Health Organization [9] estimated that telemedicine has been integrated into more than 60% of its member countries’ health care systems, proving the acceptance and use of the technology. Similarly, a Precedence Research Market Report [10] projected that the telemedicine industry will grow at a compound annual growth rate of 17.16% from 2022 to 2030 and forecast a value of US $225 billion. Telemedicine adoption differs by area; North America has led the way, thanks in part to it's technological infrastructure and legislative frameworks, but emerging countries are seeing an increase in telemedicine programs. In particular, telemedicine has played a key role in the fight against diseases within endemic regions and in the delivery of important health care interventions in some parts of Africa and Asia [11].

In Sweden, telemedicine has grown in response to progressive legislation, state-of-the-art technology, and a continued commitment to patient-centered treatment [12]. With people older than 65 years expected to comprise 20% of the population of Sweden by 2050, there is a looming lack of health care resources [13]. Sweden therefore needs to deploy telemedicine solutions quickly to meet the needs of an aging population. Telemedicine for remote monitoring and chronic disease treatment is well placed to become a key approach to dealing with this demographic shift.

The COVID-19 pandemic presented health systems all over the globe, including in Sweden, with unprecedented challenges. Prior to the pandemic, telemedicine was sufficiently flexible to address a wide range of symptoms and care requirements [14]. The pandemic period unquestionably indicated that this flexibility not only offered a solid basis for treatment but also highlighted the ability of telemedicine to adapt and scale in the face of global issues [15]. Telemedicine consultations increased dramatically in the first half of the pandemic, with some health care providers reporting an increase of more than 300% compared with the same period the year before [16]. Despite this, the postpandemic world showed that telemedicine, once looked upon as the future, is now very much a present reality, leading the way in setting the course of patient care, beyond what was conceived of in the past [17]. This new epoch provides us a beacon of hope and a new field of research as technology, medical expertise, and patient centricity converge into a medical model.

The aims of this study were to, first, elaborate on what patient symptoms prompted their use of telemedicine consultations (using a respectful approach) and explore the differences, if any, between the pre–COVID-19, COVID-19, and post–COVID-19 pandemic periods. Second, we aimed to assess differences in patterns of telemedicine consultations in the pre–COVID-19, COVID-19, and post–COVID-19 pandemic periods. Third, we aimed to analyze changes in the primary care use of telemedicine and the pattern of changes in this use in the pre–COVID-19, COVID-19, and post–COVID-19 pandemic periods. The COVID-19 pandemic was an unprecedented occurrence that necessitated telemedicine consultations and led to the widespread use of telemedicine. This study provides a critical analysis of trends and shifts in Swedish use of telemedicine consultations over time.

## Methods

### Study Design

An observational, retrospective design was used for this study. In a situation where experimentation is not controlled, researchers can observe trends or correlations without affecting the system [18]. This study involved telemedicine consultation formats and the symptoms noted during consultations in the pre–COVID-19 period of November 2018 to November 2019, the COVID-19 period of December 2019 to July 2022, and the post–COVID-19 period of August 2022 to June 2023.

### Data and Population

The population for this study was all residents of Sweden in the periods under study. Data was collected from Kry Primary Care Limited, a leading health start-up and telemedicine provider that offers insightful data for research purposes and promotes accessible health care across several countries, including Sweden, the United Kingdom, France, Germany, Norway, and Spain.

Commonly, researchers use a primary methodology for obtaining aggregated and anonymized data from Kry Primary Care’s large pool of telemedicine consultations, which conform to strict ethical guidelines and privacy regulations to ensure patient privacy. The dataset is a detailed record of all telemedicine consultations during the period of interest for this research. The data were recorded and collated by a designated research assistant in the organization and accessed after necessary approvals had been granted by the organization at different stages of verification and approval.

Kry Primary Care’s datasets are a rich resource for telemedicine and include patient demographics, clinical outcomes, and use patterns. Kry Primary Care operates through a mobile app that patients can use to remotely consult physicians, as well as psychologists and other health care specialists, removing the need for conventional physical appointments.

The data from Kry Primary Care comprise the records of text-based consultations and video meetings, as well as the symptoms diagnosed by the health care providers after the consultations. Symptoms of skin rash, poisonous animal bites, and acne were categorized as skin disorders; sore throat, cold and flu, cough, fever, diarrhea, sinusitis, and eye infections were categorized as COVID-19–like symptoms; pollen and other allergies were categorized as allergy symptoms; chlamydia, urinary tract infections, and contraceptive prescriptions were categorized as reproductive health–related issues; and general health, revisits, and prescription renewals were categorized as general health-related issues. Other conditions included sleep disorders and threadworm infections.

### Statistical Plan

Descriptive analyses of text and video consultations performed during the periods before, during, and after COVID-19 were prepared. Two-way cross-tabulation was used to summarize the symptoms and compare them before, during, and after COVID-19. These methods were used to provide an efficient summary of the trends across time periods and consultation types.

A 2-way, between-subjects, generalized linear model was used to establish significant interaction effects and main effects between the periods and meeting consultation styles. A generalized linear model offered necessary flexibility to this study given the complex nature of health care, which involves nonnormal distributions. The Tukey's HSD test was used post hoc to determine the levels of each factor that were significantly different from each other, controlling for type I errors across multiple comparisons. SPSS (version 25; IBM Corp) was used for the data analysis.

### Ethical Considerations

This research was exempted from human subjects research ethics review due to the nature of the dataset, which contains anonymized secondary data collected by Kry Primary Care. The company uses deidentified data from patients who consent to the company’s terms of service and privacy policy for research purposes. Consequently, additional informed consent was not required; likewise, no compensation was provided, as the secondary data did not involve any personal interaction and did not include access to patient-identifiable information. This secondary analysis did not need a review from an institutional review board, given that the data were collected in Sweden and the study was conducted in Sweden. According to Swedish Ethical Review Authority [19], “If you have designed a study so that no sensitive personal data or personal data about violations of the law will be received and you do not intend to process such data, no ethical review is needed.” Consequently, the authors obtained permission only from Kry Primary Care to use the data for this research.

## Results

### Patients’ Symptoms That Prompted Telemedicine Consultations

Table 1 shows that the most pronounced symptom category across all periods was general health-related issues: 186.9 of 1000 consultations before COVID-19, 1264.6 of 1000 consultations during COVID-19, and 319.2 of 1000 consultations after COVID-19; more general health-related issues were reported during COVID-19 than after COVID-19 and before COVID-19. Other symptoms that prompted telemedicine consultations included skin disorders, COVID-19–like symptoms, allergies, reproductive health–related issues, sleep disorders, and threadworm infections. There were more COVID-19–like symptoms, such as sore throat, cold and flu, cough, fever, diarrhea, sinusitis, and eye infections, during COVID-19 (314.1/1000 consultations) and after COVID-19 (110.4/1000 consultations) and fewer occurrences before COVID-19 (141.4/1000 consultations).

**Table 1.** Patient symptoms per 1000 consultations before COVID-19, during COVID-19, and after COVID-19.

	Before COVID-19, consultations per 1000	During COVID-19, consultations per 1000	After COVID-19, consultations per 1000
Skin disorders	129.4	409.0	95.7
COVID-19–like symptoms	141.4	378.9	161.0
Allergies	21.6	0.6	14.9
Reproductive health–related issues	41.9	314.1	110.4
General health-related issues	186.9	1,264.6	319.2
Sleep disorders	9.7	—^a^	—
Threadworms	15.4	4.5	—

a Not applicable.

### Differences in the Pattern of Telemedicine Consultations

The data showed that there were 2 patterns of telemedicine consultations in Sweden: text meetings and video meetings. There were more video meetings (mean 66,415.38, SD 23,737.20) than text meetings (mean 7247.73, SD 4747.52) across the 3 periods (before COVID-19, during COVID-19, and after COVID-19). There were significantly more text meetings during COVID-19 (mean 8428.31, SD 5564.29) than after COVID-19 (7965.27, SD 4747.52) or before COVID-19 (mean 3734.54, SD 2053.39). Likewise, more video meetings were recorded during COVID-19 (mean 76,977.19, SD 21,475.66) than after COVID-19 (mean 71,459.73, SD 5162.53) or before COVID-19 (38,364.15, SD 15,832.76), as presented in Table 2.

**Table 2.** Descriptive statistics for patterns of telemedicine consultations among patients in Sweden before, during, and after COVID-19.

Meeting types and periods		Meetings, mean (SD)
**Text meetings**		
	Before COVID-19	3,734.54 (2,053.387)
	During COVID-19	8,428.31 (5,564.290)
	After COVID-19	7,965.27 (1,361.069)
	Mean of Totals	7,247.73 (4,747.516)
**Video meetings**		
	Before COVID-19	38,364.15 (15,832.756)
	During COVID-19	76,077.19 (21,475.659)
	After COVID-19	71,459.73 (5,162.527)
Mean of Totals	66,415.38 (23,767.203)
**Totals**		
	Before COVID-19	21,049.35 (20,836.101)
	During COVID-19	42,252.75 (37,475.717)
	After COVID-19	39,712.50 (32,702.515)
	Mean of Totals	36,831.55 (34,265.881)

A 2-way, between-subject, generalized linear model was used to assess the main and interaction effects of period (before, during, and after COVID-19) and meeting style. As presented in Table 3, there was no statistically significant main effect of period on the number of telemedicine consultation meetings (*F*_2_=1.653; *P*=.38); in essence, the number of meetings in any period was not statistically different. The main effect of meeting type (text or video) on the number of consultations was statistically significant (*F*_1_=28.682; *P*=.03) with a large effect size (η=0.934) at a 95% CI, indicating that video meetings were significantly more frequently used for telemedicine consultations than text meetings. The interaction effect between meeting type and period was statistically significant (*F*_2_=14.723; *P*<.001) with a small effect size (η=0.22); for the pre–COVID-19 period, there were more video meetings than text meetings; likewise, during and after COVID-19, there were more video consultations than text meetings.

Pairwise comparisons of the interaction effect further proved that there was a significant increase in video meetings during the COVID-19 period compared to the pre–COVID-19 period. Similarly, there was a significant increase in video meetings during the post–COVID-19 period compared to the pre–COVID-19 period, while there was no difference in consultations between the COVID-19 and post–COVID-19 periods. Meanwhile, period did not have any significant effect on text meetings.

**Table 3.** ANOVA table of treatment means for telemedicine consultations among patients in Sweden before, during, and after COVID-19.

Source	Mean square	*F* test (*df*)	*P* value
Intercept	1.066 × 10^11^	26.86 (1)	.04
Meeting type	6.902 × 10^10^	28.682 (1)	.03
Period	4.269 × 10^10^	1.653 (2)	.38
Meeting type × period	2.583 × 10^10^	14.724 (2)	<.001

### Changes in the Primary Care Use of Telemedicine

Primary care use before, during, and after COVID-19 involved either text meetings or video meetings. During all 3 periods, the number of video meetings was significantly higher than text meetings. The mean difference was the lowest before COVID-19 (mean 34,629.62; *P*<.001) and highest during COVID-19 (mean 67,648.88; *P*<.001); it then decreased after COVID-19 (mean 63,494.46, *P*<.001), as shown in Table 4.

**Table 4.** Mean difference in number of primary care uses of telemedicine consultation among patients in Sweden before, during, and after COVID-19.

	Mean (SE) difference between video and text meetings	*P* value
Before COVID-19	34,629.615 (5,195.856)^a^	<.001
During COVID-19	67,648.875 (3,311.721)^a^	<.001
After COVID-19	63,494.455 (5,648.491)^a^	<.001

a Significant at 95% level of confidence.

## Discussion

### Overview

This study aimed to investigate symptoms that prompted Swedish patients to seek telemedicine consultations and explore the differences, if any, before, during, and after the COVID-19 pandemic. The study further assessed differences in the pattern of telemedicine consultations before, during, and after the COVID-19 pandemic and analyzed changes in the primary care use of telemedicine and patterns of change in use in these periods.

Our findings provide evidence that, during the COVID-19 pandemic, there was a pronounced increase in telemedicine consultations for general health-related problems compared to the pre–COVID-19 and post–COVID-19 periods. As telemedicine was the only available alternative to access health care in the context of the public health measures taken during COVID-19, it is possible to correlate this marked elevation in teleconsultations for general health-related issues with other recorded symptoms. This is in contrast to the substantial reduction during the post–COVID-19 period, which mirrored the winding down of strategies for adapting and stabilizing health care services. It contradicts the findings of Kambris et al [20] that the execution of lockdown guidelines, along with a fear of visiting health clinics, resulted in the relocation of health care-seeking behavior to telemedicine. Given the increased risks of exposure associated with accessing health care in person during the COVID-19 pandemic, it seems that people reached out for a virtual consultation for general concerns about their health in order to limit their possible exposure to the virus.

As expected, the number of appointments for symptoms possibly caused by COVID-19 (sore throat, cold, flu, and fever), for example, increased during the pandemic when compared to the pre–COVID-19 period, given the level of concern and awareness at the time. However, these consultations maintained a consistent presence after COVID-19 due to the enduring role of telemedicine in addressing acute, potentially infectious conditions. This finding resonates with the existing literature underscoring the surge in telemedicine use during the pandemic due to an overlap between the symptoms of COVID-19 and those of other respiratory illnesses, such as the common cold or flu [21]. While individuals were unable to distinguish COVID-19 from other respiratory infections without the help of a medical professional, they were more amenable to communicate with health care professionals through digital platforms to ensure appropriate diagnosis and guidance because these platforms provided easy access to health care and were unhindered by physical boundaries. It was thus a key finding that postpandemic use of telemedicine persisted for general health and acute conditions, possibly indicating a permanent shift in health care–seeking behavior.

Our results further showed a clear preference for video meetings over text meetings across all study periods. This shows all the more the preference for meetings between health care providers and seekers that are close to home, intuitive, and clear; video meetings can improve the precision and quality of health care delivery. The results from this study reflect earlier research showing that face-to-face communication is more effective in building rapport, developing trust, and delivering nonverbal cues [22], all of which are important in the context of health care [23]. In addition, the findings corroborate [24] that technology advancement, improved internet connection, and the acceptance of telemedicine as a competitive choice to in-person consultations have improved the use of video meetings in the health care industry. Health care providers and searchers looking to avoid traveling physical distances have made the choice of video sessions because the human connection still needs to be preserved, even in the virtual world.

Furthermore, there was an increase in text and video consultations during the COVID-19 pandemic; this is consistent with the global requirement for remote health care solutions in the face of pandemic-related limits [25]. The number of text meetings increased dramatically throughout the COVID-19 period, as compared to the pre–COVID-19 period, and further increased into the post–COVID-19 period. This trend was mirrored in video meetings, which peaked during COVID-19 and underscored the essential role that visually engaging platforms will play in maintaining health care continuity in future circumstances similar to the COVID-19 pandemic. The rise of cell phone–optimized text-based communication in the health care sector is shown by the large difference in number of text consultations between the pre–COVID-19 and post–COVID-19 periods. As suggested by the findings of Hylock and Zeng [26] and Woodward et al [27], text consultation is effective and convenient for follow-up therapy and nonemergency medical matters; patients can consult with their health care provider asynchronously without an in-person meeting and can reschedule conveniently. Importantly, the ongoing use of text- and video-based meetings after COVID-19 demonstrates that the pandemic has affected the ways in which we seek health care and that we need to expand this research to better contextualize it.

This study further found that there was no statistically significant difference in the number of telemedicine consultations. This result differs from what we expected, because previous studies have shown how effective telemedicine can be in times of medical emergency [28-30]. The lack of significant differences in the number of telemedicine consultations might be due to the digital divide; for example, demographic groups that have limited access to technology and internet services might have found it harder to take part in telemedicine consultations, resulting in bias in our data. In addition, the consistent number of consultations across the different periods may have been due to patient preferences and comfort levels with telemedicine. Some suggested that they preferred in-person visits because they liked the opportunity to speak directly to their health care provider or practitioner or because these visits gave them greater confidence in the accuracy of diagnoses, as they were made based on in-person physical examinations rather than video only. The disparity between patients’ preferences and the potential benefits of telemedicine emphasize that incorporating patient views is critical to deploying new medical technology. There seems to have been no appreciable shift in the quantity of consultations when comparing different COVID-19 stages.

Technology is a part of today’s digital age, especially in health care. By removing geographical challenges and providing flexible scheduling, video meetings make health care accessible to people with mobility difficulties or living in remote places [31]. Across all time periods in this study, some patients preferred video meetings with their physicians more than any other modality of consultation, suggesting an innate bias toward contact modalities that offer interactiveness and visual richness versus those that deemphasize the human-connection aspect of health care interactions. Several factors may have contributed to the popularity of online preferences. The innate propensity toward more interactive consultation modalities may be a desire to improve health care providers’ and patients’ ability to communicate and interact with one another. Moreover, robust and efficient health care service delivery is possible with video meetings, as they allow real-time engagement and visible facial expressions and body language clues [32].

### Limitations

The results of this study should be interpreted with caution because of the presence of a few limitations. First, the data came only from telemedicine consultations in Sweden, and may not be generalizable to other locations because of confounding factors such as differences in health care systems and patient preferences outside of Sweden. As a result, the findings should not be generalized without proper qualification; this opens an opportunity for further studies to replicate the study with a wider geographical scope.

Second, the study data were collected retrospectively from telemedicine consultations, which may have resulted in errors or omissions in the results due to variability in the accuracy and completeness of symptoms and diagnoses recorded through consultations. In future research, this limitation can be mitigated, for example by comparing the recorded symptoms and diagnoses in the telemedicine consultations with other sources of medical data, such as electronic health records, patient self-reports, or follow-up consultations, while juxtaposing the records against patient feedback.

Third, this study was limited in its ability to explain the causes of the observed variances and similarities in telemedicine consultations across time. Further research could investigate the socioeconomic factors that may have influenced patients to use telemedicine during the COVID-19 outbreak.

Fourth, some text meetings were converted to video meetings as a function of necessity in specific cases, but the dataset did not specify which meetings were converted from text to video consultations. Hence, there is a likelihood that this could have affected the results.

### Conclusions

This study examined symptoms for telemedicine consultations in Sweden and their trends before, during, and after the COVID-19 pandemic. During COVID-19, general health-related teleconsultations increased, perhaps because of public health concerns and a desire to minimize exposure. During the pandemic, there was a surge in COVID-19 consultations which demostrates that telemedicine can help manage symptoms such as sore throat and fevers. The results show the preference of the sample for video meetings over text meetings, which emphasizes the need to prioritize the visual and interactive aspects of communication in health care delivery. Socioeconomic considerations may have influenced patient participation in telemedicine during the COVID-19 pandemic, based on these findings. More research on public perceptions and attitudes affecting video preferences in telemedicine may help explain post–COVID-19 health care–seeking habits. Finally, further studies could examine telemedicine adoption barriers, such as the digital divide and access to technology and internet services, in an effort to increase telehealth service use for all demographics.
